# Probing charge transfer between molecular semiconductors and graphene

**DOI:** 10.1038/s41598-017-09419-3

**Published:** 2017-08-25

**Authors:** Aleksandar Matković, Markus Kratzer, Benjamin Kaufmann, Jasna Vujin, Radoš Gajić, Christian Teichert

**Affiliations:** 1 Institute of Physics, Montanuniversität Leoben, Franz Josef Strasse 18, 8700 Leoben, Austria; 2 Graphene Laboratory of Center for Solid State Physics and New Materials, Institute of Physics, University of Belgrade, Pregrevica 118, 11080 Belgrade, Serbia

## Abstract

The unique density of states and exceptionally low electrical noise allow graphene-based field effect devices to be utilized as extremely sensitive potentiometers for probing charge transfer with adsorbed species. On the other hand, molecular level alignment at the interface with electrodes can strongly influence the performance of organic-based devices. For this reason, interfacial band engineering is crucial for potential applications of graphene/organic semiconductor heterostructures. Here, we demonstrate charge transfer between graphene and two molecular semiconductors, parahexaphenyl and buckminsterfullerene C_60_. Through *in-situ* measurements, we directly probe the charge transfer as the interfacial dipoles are formed. It is found that the adsorbed molecules do not affect electron scattering rates in graphene, indicating that charge transfer is the main mechanism governing the level alignment. From the amount of transferred charge and the molecular coverage of the grown films, the amount of charge transferred per adsorbed molecule is estimated, indicating very weak interaction.

## Introduction

Graphene has a significant potential to be used as a new transparent conductive electrode material in flexible and wearable electronics, optoelectronics, and energy applications^1, 2^. In addition to high transparency, high mechanical strength, flexibility, thermal as well as chemical stability, and ease of functionalization, there are also crucial advantages of graphene as an electrode material in organic electronics. These are mainly based on the favorable band alignment with many organic semiconductors and on their impeccable growth morphologies on graphene, which relies on the van der Waals (vdW) nature of the interface^3^. As a consequence, these heterostructures exhibit low injection barriers, high charge extraction and injection efficiencies with electronic decoupling and preservation of the intrinsic functionality of the molecular crystals at the interface^4–19^. Hence, graphene is employed as a carrier injection layer between organic semiconductors and metallic contacts^4^ and as vdW electrode^5–7^. Efficient charge separation in graphene/organic semiconductors photo-transistors^15^ as well as organic light emitting diodes^20–23^ have been demonstrated. Moreover, heterointerfaces between organic semiconductors and other two-dimensional (2D) materials – like hexagonal boron nitride and MoS_2_ – have been realized, enhancing existing and enabling new functionalities in organic electronics based devices^7, 13, 18, 24, 25^.

Interfacial band engineering–through charge transfer and band alignment–is crucial for potential applications of graphene-organic semiconductor heterostructures. Doping of graphene by charge transfer with organic molecular layers has been investigated using Kelvin probe force microscopy and electrical measurements of graphene field effect devices^9, 10^. Using a gated graphene field-effect device enables precise control of graphene’s Fermi level position^26^, which affects charge transport through the interface^14, 23^. This allows even tuning the molecular orbitals at the interface^27^. Furthermore, *in-situ* sheet resistance measurements combined with ultraviolet photoelectron spectroscopy have been used to investigate charge transfer between graphene and Cs_2_CO_3_ in an organic matrix^22^, where the formation of a large interface dipole and n-type doping of graphene were observed. In general, materials with a work function (Φ) substantially lower than that of graphene are needed to achieve n-type doping, as was also demonstrated by deposition of potassium and ZnO^23, 28^. Recently, interfaces between graphene and metal oxides have been investigated as effective hole-injection layers in organic light emitting diodes^20, 21^, exhibiting p-type doping of graphene through charge transfer and formation of an interfacial dipole.

Owing to the unique density of states of graphene^26^ and the exceptionally low electrical noise^29^, field effect devices can be utilized as extremely sensitive potentiometers for probing charge transfer between graphene and adsorbed species. This was used even to detect individual adsorption/desorption events of gas molecules as NO_2_ on micrometer-scale devices^30^. Moreover, *in-situ* electrical characterization was employed to reveal the relation between SiO_2_ and the effective p-type doping of graphene under ambient conditions^31^, showing the necessity of both, water and oxygen in this process.

Nonetheless, the high sensitivity of graphene devices goes along with a lack of selectivity. The effects of marginal remnants, arising from the device preparation (degassing, annealing, purging, exposure to ambient conditions) often mask the charge transfer effect that is actually under investigation. For this reason, *in-situ* electrical characterization is needed which strictly avoids further sample treatment and exposure to ambient atmosphere, especially in the cases when charge transfer is expected to be small. Yet, a study of charge transfer between molecular crystals and graphene by *in-situ* electrical measurements with simultaneous capability of tuning graphene’s Fermi level position and type of majority carriers is greatly lacking.

In this study, we examine charge transfer between graphene and two molecular crystals: para-hexaphenyl (6P)^32, 33^, a wide HOMO-LUMO gap, intrinsic organic semiconductor with well matching Φ to graphene^34, 35^; and buckminsterfullerene C_60_, an n-type semiconductor with Φ over 1 eV larger than that of graphene^36, 37^. The organic thin films (0.5–10.0 monolayers (ML)) have been prepared using a hot wall epitaxy (HWE) setup^38^ equipped with electrical contacts to the sample in order to enable *in-situ* electrical characterization. As substrates, field effect transistors (FETs) with an exfoliated graphene channel have been used.

We study *in-situ* the effect that deposited molecular crystals have on the transfer curves of graphene FETs, and thus probe the formation of the interfacial dipole. The setup allows us to control graphene’s Fermi level position prior (and during) the growth experiments. The estimated charge transfer per adsorbed molecule deduced from the graphene’s Fermi level shift and the molecular coverage indicate a very weak interaction. Furthermore, the same setup has been used for desorption experiments with 6P, showing that the charge transfer process is completely reversible upon 6P desorption.

## Results and Discussion

### Introducing the setup

**Figure 1:**
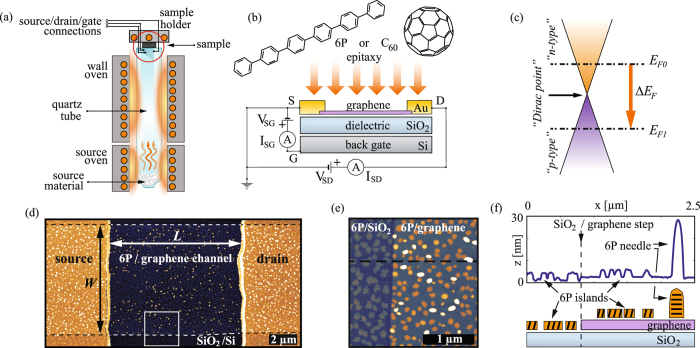
Experimental setup: (**a**) layout of the HWE setup. The scheme of the sample (marked by an orange circle) is enlarged in (**b**) showing upside-down the graphene FET with the *in-situ* DC electrical characterization scheme. (**c**) Graphene’s dispersion relation. E_*F*0_ and E_*F*1_ indicate the position of the Fermi level (with respect to the Dirac point) before and after epitaxy, while ΔE_*F*_ indicates the shift of graphene’s Fermi level. (**d**) AFM topography image of one of the devices used in the study after the deposition of a sub-monolayer of 6P (*z* scale 50nm). Dashed lines in (**d**) highlight the rims of the graphene flake. *L* and *W* mark channel length and width. (**e**) Area exhibiting a step-edge between graphene and SiO_2_, indicated in (**d**) by a square and rotated by 90° with respect to (**d**) (*z* scale 25 nm). (**f**) A height cross section along the dashed line in (**e**) is shown (top), with a corresponding layout of the structure (bottom).

The custom-built HWE chamber used in this study has three electrical contacts attached to the sample holder, which allow to probe and tune electrical properties of the samples prior and during the growth. A layout of the growth chamber is illustrated in Fig. 1a. A schematic representation of a graphene FET is shown in Fig. 1b, also indicating the connections for the *in-situ* electrical measurement setup. Graphene films exhibit trace amounts of photoresist contaminations from FET fabrication^39^. These residues were observed to have similar impact on the morphology of the grown films as in the case of wet-transferred chemical vapor deposited graphene^40^.

From the transfer curves of graphene FETs (I_*SD*_(V_*SG*_)) it is possible to recalculate the position of graphene’s Fermi level, both prior and after the growth (E_*F*0_ and E_*F*1_) as illustrated in Fig. 1c (see supplementary information for more details). The charge neutrality point (CNP) of graphene was found not to be exactly at V_*SG*_ = 0 V prior to the growth experiments. This has been attributed to the unintentional doping that results mainly from the trapped interfacial layer between graphene and SiO_2_, exposure to ambient water vapor, and lithography residues, thus giving a different CNP value for each sample^31, 39^. For this reason, performing *in-situ* measurements of the charge transfer during the growth is essential to eliminate all other contributions (degassing, annealing, and exposure to ambient) which would affect the transfer curves of graphene in a similar way (see supplementary information for the details on the pre-treatment of graphene FETs). Figure 1d shows an atomic force microscopy (AFM) overview topography image of one of the devices covered with ~0.8 ML of 6P. A magnified region of the channel rim is shown in Fig. 1e. The height cross-section of Fig. 1e and the scheme of the structure are presented in Fig. 1f.

### 6P growth experiments

**Figure 2:**
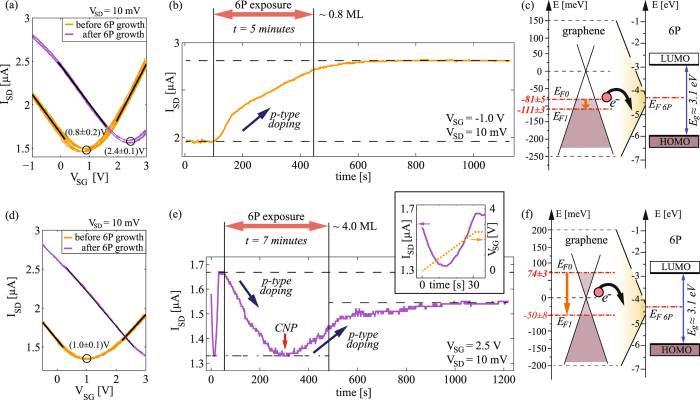
6P growth experiments: (**a**) Transfer curves (five subsequent forward and backward V_*SG*_ sweeps) of a graphene field-effect device directly before and after deposition of ~0.8 ML of 6P. (**b**) I_*SD*_ as a function of time during the deposition, starting from p-type graphene. (**c**) Band diagrams of graphene (left) and 6P (right), prior to interaction. (**d**–**f**) Analogue results for another device, on which the growth was started from n-type graphene and ~4 ML of 6P were grown. Inset of (**e**) shows an initial V_*SG*_ ramp (and corresponding I_*SD*_) used to bias the device prior to growth.

Figure 2a shows transfer curves of a graphene FET measured at 2 × 10^−6^ mbar (300 K) in the growth chamber, directly prior and after the growth of ~0.8 ML of 6P. The device was slightly p-doped with a CNP atV_*CNP*_ = (0.8 ± 0.2) V, giving E_*F*_ = −(56 ± 8) meV. The Fermi level was kept below the neutrality point (E_*F*0_ = −(81 ± 5) meV), within the linear part of the transfer curve by setting V_*SG*_ = −1V during the growth experiments. The *in-situ* measurements of I_*SD*_ during the growth (Fig. 2b) reveal that upon exposure of the device surface to 6P I_*SD*_ immediately starts to increase. Considering that graphene was initially p-type, an increase in the current indicates further p-doping by the adsorbed molecules. Schematic representations of the band diagrams of graphene and 6P are shown in Fig. 2c, indicating the estimated positions of the graphene’s Fermi level prior and after the growth. The different scale (a factor of 20) for the energy axis between graphene and 6P is used to make graphene’s Fermi level shift visible.

Solid lines in Fig. 2a represent linear fits used to estimate the field-effect mobilities^41^. Devices used in this study had field-effect electron and hole mobilities in the range of 3000–6000 cm^2^ V^−1^ s^−1^, which is in a good agreement with the data reported for exfoliated graphene on SiO_2_^30^. Interestingly, the main difference between the transfer curves prior and after the deposition of 6P is a parallel shift. Only minor changes in the minimal value of I_*SD*_ and in the field-effect mobilities (slopes of the curves) were detected in all growth experiments. This fact unambiguously proves that the deposited thin layers of the molecular crystals did not affect scattering rates in graphene. Thus, the doping mechanism is mainly based on charge transfer between graphene and the adsorbed molecules.

Figure 2d–f shows analogue results to those presented in Fig. 2a–c, obtained from another device on which the growth was started from n-type graphene and ~4 ML of 6P were grown. As shown in Fig. 2d (orange curves), the device was unintentionally p-doped prior to the growth experiments, with CNP at V_*SG*_ = (1.0 ± 0.1) V. In order to ensure that graphene’s Fermi level is above the CNP at the beginning of the growth experiment, a constant voltage V_*SG*_ = 2.5 V was applied during the growth, thus giving the initial position of the Fermi level of E_*F*0_ = (74 ± 3) meV. The inset in Fig. 2e highlights the first 40 seconds of the diagram, during which V_*SG*_ was ramped from 0 V to 2.5 V prior to 6P deposition. As the gate voltage increases, the I_*SD*_ curve changes from p-type graphene–through CNP–to n-type graphene. Then, the device was exposed to 6P vapor and the current immediately decreased. Since the experiment started from initially n-type graphene, a decrease in current (at constant V_*SG*_) indicates again p-type doping by the adsorbed molecules. Interestingly, the minimal value in current which was reached during the growth experiment matches exactly the minimal value reached by sweeping V_*SG*_. This indicates that adsorbed molecules lower the Fermi level of graphene from n-type–through the neutrality point and into p-type–where further p-type doping is seen as an increase in the current. This was confirmed by measuring transfer curves after the growth (Fig. 2d). The fact that the electrostatic gating and 6P deposition yield the exact same minimal value of I_*SD*_ further supports that charge transfer doping at the 6P/graphene interface is the main mechanism through which the adsorbed molecules affect the electrical properties of the device.

### 6P desorption experiment

The reversibility of the charge transfer process was demonstrated by desorption experiments, for the case of 6P. The experiments were carried out in the same HWE chamber, where previously grown 6P films were annealed (at 415–425 K) in high vacuum for an extended period of time, hence releasing the molecules from the surface of the graphene FETs. The same *in-situ* electrical characterization has been carried out as for the growth experiments, with the slight difference that the transfer curves after annealing were only measured once the sample has reached again 300 K.

**Figure 3:**
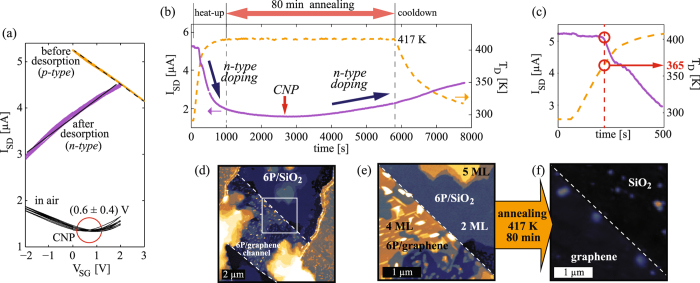
6P desorption experiment: (**a**) Set of transfer curves of a device initially covered with ~4 ML of 6P (Fig. 2(d–f)), in high vacuum before and after desorption of 6P, and in air after desorption of 6P. (**b**) Current through the device during annealing (solid line) and the corresponding T_*D*_ (dashed line). (**c**) Enlarged initial 500 s of (**b**) showing the desorption temperature for the 6P thin film on graphene. (**d**) AFM topography of the device prior to desorption experiment, *z* scale 60 nm. (**e,f**) Enlarged AFM images of a graphene/SiO_2_ edge (marked by a square in (**d**)) before and after annealing (*z* scale 30 nm).

In Fig. 3, we present the data obtained from the desorption experiment of the device shown in Fig. 2d–f. Between the growth and desorption experiments, the morphology of the 6P film was characterized by AFM under ambient conditions, thus inevitably exposing the graphene/6P interface to water vapor, enhancing p-type doping of graphene^31^. For comparison, we provide in Fig. 3a transfer curves of the device before and after desorption of ~4 ML of 6P, as well as in ambient air after the desorption. Annealing in high vacuum was carried out by heating the sample up to 417 K for 80 minutes, followed by a slow cool-down to room temperature. During annealing of the device, I_*SD*_ was measured and correlated to sample temperature T_*D*_, as shown in Fig. 3b. Since the graphene was initially p-doped, a reduction of I_*SD*_ indicates n-type doping demonstrating the reversibility of the charge transfer mechanism. The minimal value of the current reached by annealing was slightly higher than for the case of electrostatic gating (Figs 2d and 3a), since in this case the sample was at T_*D*_ = 417 K, which certainly affects both, the scattering rates in graphene and the serial resistance. After cool-down, transfer curves were again measured, showing n-type behavior (Fig. 3a). The field-effect mobilities of graphene were not significantly affected by the annealing and desorption of the molecules.

Figure 3c shows the magnified heat-up step of the annealing process (the first 500 s of Fig. 3b). Initially, as the device heats up, almost no change in the current is detected. This is the case until T_*D*_ reaches (365 ± 5) K, then, a rapid reduction in the current sets in, which is attributed to 6P desorption. This might be useful to determine the desorption temperature of organic semiconductors from graphene. However, both molecular rearrangement prior to desorption and the heating rate could affect the desorption temperature^42^.

AFM was used to monitor the amount of material removed from the surface by annealing. An overview of the device prior to thermal desorption is presented in Fig. 3d. Figure 3e–f compares AFM images of an area at the channel edge (indicated by the white square in Fig. 3d) before and after annealing. The same *z* scale for both images is used to highlight that the majority (over 95%) of the material was successfully removed by annealing for only 80 minutes. Extended annealing times or higher annealing temperatures would entirely remove the 6P, however, this could also affect the serial resistance of the thin gold electrodes and was therefore avoided.

### C_60_ growth experiments

**Figure 4:**
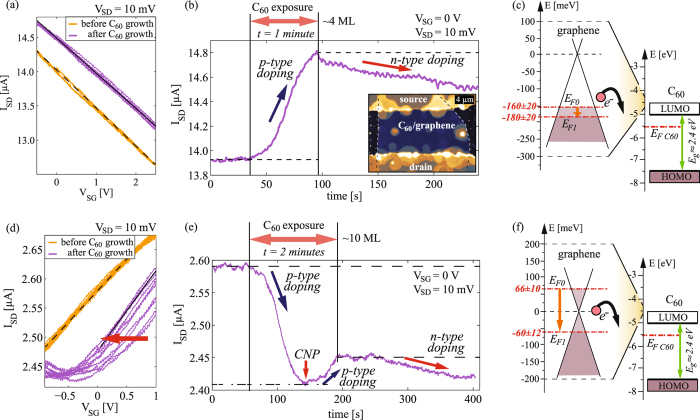
C_60_ growth experiments: (**a**) Transfer curves (five subsequent forward and backward V_*SG*_ sweeps) of a graphene field-effect device immediately before and after deposition of ~4 ML of C_60_ and (**b**) I_*SD*_ as a function of time during the deposition starting from p-type graphene. The inset of (**b**) shows the AFM topography of the sample after the growth. (**c**) Band diagrams of graphene (left) and C_60_ (right) prior to interaction. The energy scales between graphene and C_60_ have a factor of 20 difference. (**d–f**) Analogue results for another device, on which the growth was started from n-type graphene, and ~10 ML of C_60_ were grown. An arrow in (**d**) indicates the direction of the shifting of the subsequently measured transfer curves.

Using the same method as for 6P, charge transfer at the interface between graphene and C_60_ was also investigated. Figure 4 provides results from the *in-situ* electrical measurements during the growth of C_60_. Data for two devices are presented in the same manner as for 6P (Fig. 2). Figure 4a shows transfer curves of an intrinsically p-doped device prior to growth, V_*CNP*_ = (7.0 ± 1.5) V. Due to strong initial intrinsic p-doping of the sample, the CNP could not be reached within the accessible range of V_*SG*_ (without risking electrostatic breakdown of the SiO_2_). The position of the CNP was estimated considering the value observed in air prior to degassing and annealing of the device. Hence, there is a larger uncertainty of the exact initial position of the Fermi level (E_*F*0_ = −(160 ± 20) meV). However, this does not affect the shift of the transfer curves and the amount of transferred charge introduced by the adsorbed molecules, which can still be estimated from Fig. 4a. During the growth, I_*SD*_ was recorded as a function of time (Fig. 4b). Starting from p-type graphene, an increase in current upon exposure to C_60_ indicates further p-doping. The inset in Fig. 4b is an AFM topography image of the device with ~4 ML of C_60_. In Fig. 4c, a scheme of the graphene and C_60_ band structures is presented with different energy scales for graphene and C_60_ visualizing the graphene’s Fermi level shift.

In analogy to Fig. 4a–c, data of another device (initially slightly n-doped) is shown in Fig. 4d–f with ~10 ML of C_60_ grown. Steady p-type doping of graphene with C_60_ exposure was also observed in this case leading to a lowering of graphene’s Fermi level from the n-type– through the neutrality point– into the p-type regime. This can be well observed by following the *in-situ* measurements of I_*SD*_ in Fig. 4e.

Interestingly, in the case of C_60_, I_*SD*_ did not saturate at a certain value immediately after the growth, as it was the case for 6P. Yet, after C_60_ deposition I_*SD*_ kept changing, indicating n-type doping of graphene (see Fig. 4b,e). The value for I_*SD*_ saturated just several minutes after the growth has been stopped. An unstable level of doping was also observed in the transfer curves after the growth (shown in Fig. 4d). There, successive transfer curves are shifted to lower voltages as indicated by the arrow. Again, the lateral shift of the transfer curves indicates that the underlying process is mainly based on the charge transfer. The direction of the shift (marked by a red arrow in Fig. 4d) reveals that electrons were transferred back to graphene, introducing n-type doping. The observed doping instability in graphene after C_60_ exposure is most likely caused by interaction with the remaining oxygen in the HWE growth chamber. Diffusion of oxygen into C_60_ films was previously shown to affect the interfacial dipole formed between C_60_ and highly oriented pyrolytic graphite^37^ and to deteriorate electrical conductivity of C_60_ based devices^43^.

### Band diagrams and charge transfer

**Figure 5:**
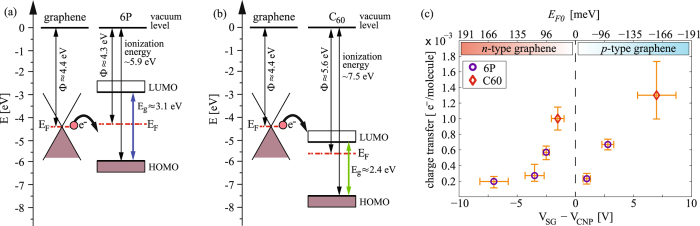
Band diagrams and charge transfer: (**a,b**) band diagrams prior to establishing the equilibrium (equal vacuum levels) for graphene/6P and grapene/C_60_, respectively. (**c**) Charge transfer per molecule as a function of the position of CNP prior to the growth, for all the devices used in this study. Circles represent the data for 6P/graphene and diamonds for C_60_/graphene interfaces.

Transfer of electrons from graphene–and consequent p-type doping of graphene by both 6P and C_60_–is self-evident when the difference between the Φ of graphene (≈4.4 eV)^44^ and the ionization energy of the molecular crystals is considered. In both cases, the Fermi level in graphene is expected to be at least 1 eV above the highest occupied molecular orbitals. Figure 5a,b shows band diagrams for graphene/6P and graphene/C_60_ prior to interaction (considering equal vacuum levels). The data for the ionization energies, Φ, and band gaps of 6P and C_60_ were taken from ultraviolet photoelectron spectroscopy (UPS) of bulk molecular crystals and thin films deposited on highly oriented pyrolytic graphite^35–37, 45^. As the interface is formed, electrons are transferred from graphene into the growing molecular crystals. Considering the low density of states of both, 6P and C_60_, and the small amount of material in the grown films (less than 10 nm), Fermi level alignment in the ordinary sense (bulk interfaces) is most likely not possible and would require significantly thicker films^37^.

Since the shift of the CNP is known, it is possible to estimate the number of transferred electrons from graphene. The detailed morphology of the grown films revealed by AFM (see supplementary information) together with the most probable crystal phases of both 6P and C_60_ (Baker structure and face centered cubic, respectively), allow an estimation of the number of deposited molecules. From these two values, the average charge transfer per adsorbed molecule can be calculated. A compilation of the estimated charge transfer per adsorbed molecule as function of the CNP position (initial Fermi level position) is provided in Fig. 5c. Error bars in the *x* axis indicate the uncertainty of the initial CNP position, while error bars in the *y* axis mainly originate from the uncertainty of the total volume of the molecular crystals grown on the device surfaces (measured by AFM). The obtained charge transfer per adsorbed 6P molecule was found to be ~4.7 × 10^−4^ electrons/6P, and for the case of C_60_ somewhat higher values of ~1.1 × 10^−3^ electrons/C_60_ were deduced. No significant change of the charge transfer was observed within the available range of the applied gate voltage (initial position of graphene’s Fermi level), and the observed differences between individual devices are likely related to sample-to-sample variations.

Similar results were obtained for the charge transfer at the graphene/pentacene interface measured by UPS^6^ and for the graphene/C_60_ interface measured by scanning tunneling spectroscopy and transport characteristics^11, 46^, revealing weak interaction and small charge transfer from graphene. Vertical heterostructures using a graphene/C_60_ interface have shown slight downshifts of graphene’s Fermi level upon C_60_ deposition^14^. In addition, charge transfers between epitaxial graphene and gold, antimony, and bismuth have somewhat larger values^47^, in the order of 2–10 × 10^−3^ electrons taken from graphene per adsorbed metal atom. Very recently, molecules (as acetone and toluene) trapped at the interface between graphene an SiO_2_, and the influence of arrangement of C_60_ on graphene and the role of the supporting substrate on the charge transfer have been investigated^48, 49^, in both cases exhibiting charge transfer in the order of ~10^−3^ electrons per molecule.

The fact that over thousand molecules are needed to extract only one electron from graphene raises the question of the nature of the charge transfer mechanism. The charge transfer could either be fractional or integer^50, 51^. Fractional charge transfer has been reported for molecules adsorbed on clean metal surfaces, usually through weak chemisorption or covalent bonding^50–52^ resulting in an excess charge homogeneously distributed among the molecules, yielding an effective fractional charge per each molecule. As an alternative scenario, the integer charge transfer was reported for weakly interacting interfaces like passivated metal surfaces^50, 51^. In this case, electrons tunnel from the metal and are localized only on some molecules, while the others remain electrically neutral. In this study, fractional charge transfer is less likely due to the vdW nature of the interface. Moreover, measurements of the source-drain current during the growth did not show significant changes between the first and several subsequent layers, for the case when multi-layers are grown. This observation further indicates that the charge transfer mechanism is more likely an integer than a fractional one.

## Conclusions

In summary, a weak electronic interaction through charge transfer between graphene and molecular semiconductors (6P and C_60_) was observed. As the interface forms, less than 10^−3^ electrons per molecule are taken from graphene, consequently lowering graphene’s Fermi level and introducing p-type doping of graphene. This result was found not to be dependent on the initial type of the majority carriers in graphene (initially p- or n-type), confirming that band alignment is more important than the type of doping when considering interfacial band engineering with graphene. Moreover, the fact that the adsorbed molecules have mainly affected the ambipolar transfer curves of graphene field-effect devices through lateral shifts unambiguously proves that the scattering rates in graphene were not affected by the adsorbed molecules. This confirms that the interaction occurs principally through charge transfer and formation of an interfacial dipole, with further indications that integer charge transfer occurs in these systems.

## Methods

### Fabrication of graphene field effect devices

Graphene flakes were prepared by micromechanical exfoliation of kish graphite. Flakes were deposited on highly doped Si substrates (less than 0.01 Ω cm), serving as a back-gate electrode (G), with (80 ± 2) nm thin layer of dry thermal SiO_2_, which acts as a gate dielectric and enhances optical contrast of the flakes. Single-layer graphene flakes were selected by optical microscopy and were checked for any contaminations, wrinkles, and cracks by AFM, prior to the deposition of the electrodes. Source (S) and drain (D) top contacts were made by positive mask UV photolithography, with a (20–30) nm thick gold layer. Channel length was ~10 *μ*m, and width (*W*) was (5–30) *μ*m (depending on the shape of the flakes).

In order to have stable transfer characteristics of the devices and to ensure reproducibility of the data, all samples were left to degas in high vacuum (≈1 × 10^−6^ mbar) for 12 hours, followed by annealing in high vacuum prior to the growth of molecular crystals. More details on the pre-treatment of the graphene FETs is given in the supporting information, where 6P crystallites are also grown at elevated T_*D*_ (365 K) in order to accent the impact of the surface contaminations on the growth of the molecules.

### Electrical measurements and field-effect mobility estimates

Electrical measurements were carried out within an HWE chamber, using a Keithley 2636 A SYSTEM SourceMeter. Voltages between S and D (V_*SD*_) and S and G (V_*SG*_) were applied, and the currents between S and D (I_*SD*_) and S and G (I_*SG*_) were measured. To avoid electrostatic breakdown of the SiO_2_, I_*SG*_ was monitored and set not to exceed 1 nA. All transfer curves I_*SD*_(V_*SG*_) were measured at room temperature in either high vacuum or ambient atmosphere. Five subsequent transfer curves were measured each time to ensure reproducibility, with ΔV_*SG*_ = 0.01 V and time steps of 200 ms. For *in-situ* measurements of I_*SD*_, the time step was set to 500 ms. The sample temperature (T_*D*_) was kept constant at (300 ± 2) K in order to exclude other potential contributions to the charge transfer process. The position of the Fermi level in graphene was calculated considering a parallel plate capacitor between graphene and SiO_2_ and taking the Fermi velocity of electrons in graphene as 10^6^ m/s (see supplementary information for more details). Field effect mobilities were extracted considering the diffusive regime of charge transport (*L* = 10 *μ*m). ΔI_*SD*_/Δ V_*SG*_ was obtained from the linear parts of the transfer curves. Serial resistances (R_*S*_) of the devices (mainly arising from thin Au pads) were measured for each device batch by Kelvin probe force microscopy measurements of the voltage drop between S and D. R_*S*_ was found to be between 20% and 30% of the total device resistivity. It is worth to mention that only the absolute values for the field-effect mobilities are affected by R_*S*_.

### Hot wall epitaxy setup

Thin films of molecular semiconductors were grown using a home-built HWE setup with electrical connections to the sample holder that allow *in-situ* electrical measurements. The growth was carried out in high vacuum (base pressure of 1 × 10^−6^ mbar) with fix source (T_*S*_) and wall (T_*W*_) temperatures, for 6P T_*S*_ = 508 K, T_*W*_ = 518 K, and for C_60_ T_*S*_ = 623 K, T_*W*_ = 638 K.

### AFM measurements and molecular semiconductors film morphologies

The morphology of the samples was investigated employing an Asylum Research MFP-3D AFM system operating under ambient conditions. Olympus AC160TS probes were used with typical force constants of 20–80 N/m and tip curvature radii of 5–7 nm. AFM topography images of the samples were processed using the open source software Gwyddion (version 2.38). The thickness of the molecular crystals was estimated considering the total volume on the device active area and presented as an equivalent in complete monolayers (ML) of the bulk structure (in the case of 6P considering up-right standing molecules that form island-like crystallites) of the molecular crystals, since an ideal layer-by-layer growth was not observed. In the considered ranges of the growth temperatures, only the monoclinic *β*-phase of 6P crystallites is expected (the Baker structure) and the fcc structure of C_60_. The morphology of C_60_ films indicated layered growth (step edge height of ~0.8 nm), while 6P films were found to consist of both, islands of up-right standing molecules (~2.6 nm) and needles with flat-laying molecules (taller than 10 nm).

### Data availability statement

The datasets generated during and/or analysed during the current study that are not included in this published article (and its supplementary information files) are available from the corresponding author on reasonable request.

## Electronic supplementary material

Supplementary Information
