# Supplementary material for: Probing charge transfer between molecular semiconductors and graphene
Source: Sci Rep. 2017 Aug 25;7:9544. doi: 10.1038/s41598-017-09419-3 (PMC5572701; doi:10.1038/s41598-017-09419-3)
Supplement: Supplementary file 1 — Supplementary Information [file 41598_2017_9419_MOESM1_ESM.pdf]

Supplementary information:
Probing charge transfer between molecular semiconductors and graphene

Aleksandar Matković[1], Markus Kratzer[1], Benjamin Kaufmann[1], Jasna Vujin[2], Radoš Gajić[2], and Christian Teichert[1,*]

[1] Institute of Physics, Montanuniversität Leoben, Franz Josef Strasse 18, Leoben, 8700, Austria
[2] Institute of Physics, University of Belgrade, Pregrevica 118, 11080 Belgrade, Serbia
[*] mail to: teichert@unileoben.ac.at

Influence of the device annealing in high vacuum on the electrical properties of graphene.

As-fabricated graphene FETs experience different unintentional doping, resulting in a different position of the Fermi level (different position of the CNP in the transfer curves). This effect arises from the species trapped at the interface between graphene and SiO_2 and from unintentional contaminations of the top surface of graphene (mainly arising from the residues of UV photolithography process). In order to have doping levels as comparable as possible between the different devices and to have stable transfer curves of graphene FETs, the devices were annealed in high vacuum ($\sim 1\times10^{-5}$ mbar) prior to the growth of molecular semiconductors. The annealing temperature was varied in a range between 400 K and 450 K, and the devices were kept at this temperature for 30 min to 60 min. Within these ranges of the annealing parameters, very similar effects on the electrical properties of most of the devices were observed.

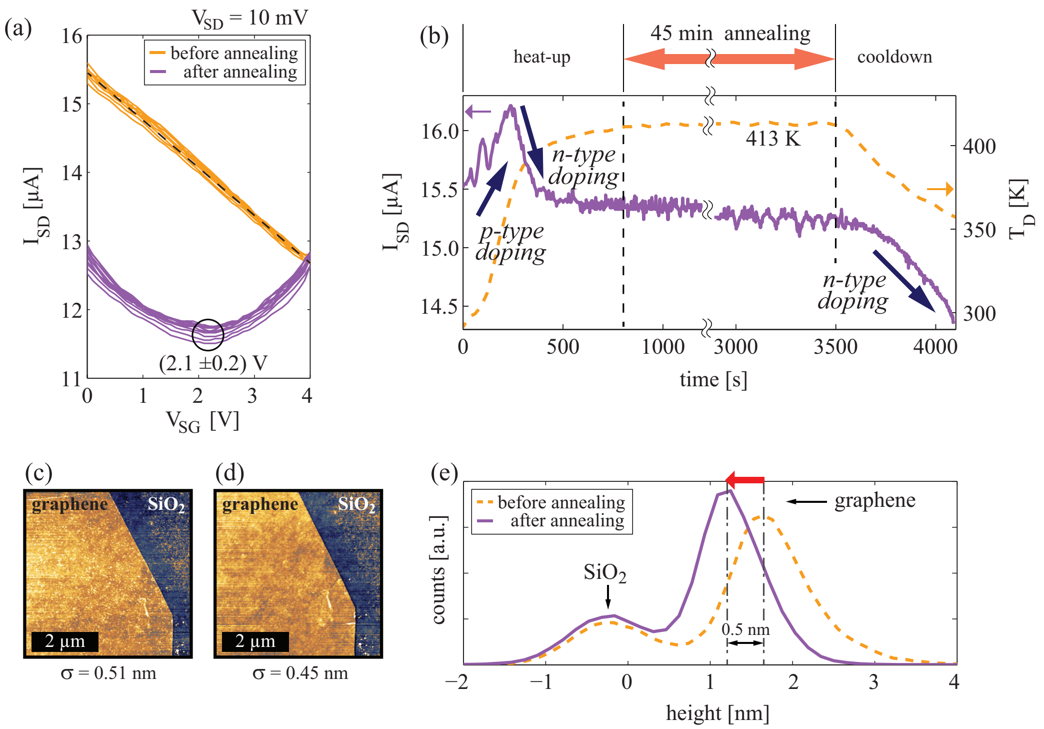

Figure S1. Influence of the device annealing in high vacuum on the electrical properties of graphene. (a) transfer curves of a graphene FET prior and after annealing in high vacuum. (b) *in-situ* measurements of I_{SD} during the annealing step (solid line) and correlated T_D (dashed line). During the annealing, the back-gate electrode was grounded and V_{SD} was fixed to 10 mV. (c) and (d) respectively show AFM topography images of the rim of the graphene channel prior and after the annealing (also indicating rms roughness σ of graphene), while (e) shows the overlayed height histograms of (c) and (d).

Figure S1 presents the effects of the annealing in high vacuum on one of the graphene devices used in this study. As can be seen from Fig. S1a, the amount of unintentional doping is reduced allowing the CNP to be reached within the safe scanning range of V_{SG}. However, the device still remains slightly *p*-doped (E_F = -(88 ±5) meV). A similar trend of Fermi level shifts was observed for most of the samples. Furthermore, the height of the graphene/SiO_2 step edge was found to decrease by

(0.3-0.6) nm upon annealing. The root mean square surface roughness of graphene also decreased upon annealing by ∼10%, both indicating the removal of the surface contamination layer.

Unfortunately, the annealing did not always result in more stable or less doped devices. After the annealing, some of the devices were even further doped, or had unstable transfer curves (not repeating subsequent transfer curves or even forward and backward sweeps). These devices were not used for the growth experiments. The negative effect of the annealing in some cases is most likely related to releasing of surface contaminations that were localized near the device, which then contaminated the surface of the channel.

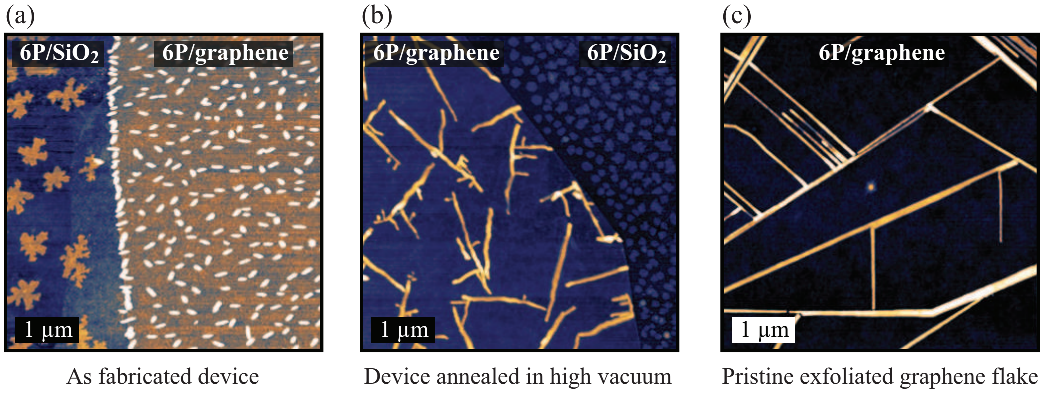

Figure S2. Influence of device annealing on the growth morphologies of 6P. 5×5 μm^2 AFM topography images (*z* scale 25 nm) of 6P crystallites grown on: (a) as fabricated graphene device, (b) device annealed in high vacuum, and (c) pristine exfoliated graphene flake. 6P grown at T_D = 365 K with a surface coverage of ∼0.6 ML in all three cases.

Influence of the surface contaminations and annealing on the growth morphologies of 6P.

In order to highlight the impact that the residues remaining on the surface of the devices have on the growth of molecular semiconductors, 6P was grown at T_D = 365 K with a surface coverage of ∼0.6 ML. This chosen T_D is higher than the ones used in the main manuscript (300 K), resulting in the growth of larger crystallites and thus emphasizes the impact of surface contaminations on nucleation and growth. As shown in Fig. S2, three different graphene surfaces were used: an as-fabricated graphene device with top surface contaminated with UV photolithography residues, a device annealed in high vacuum (the same device is also shown in Fig. S1), and a pristine as-exfoliated graphene flake which serves as a representative for the uncontaminated surface. In all cases, 6P molecules lay flat on graphene, forming needle like crystallites with their herringbone layers perpendicular to the surface of graphene. The striking difference in the nucleation density of the crystallites depending on the surface treatments clearly reveals the necessity of the annealing in order to ensure good contact between the molecules and graphene and to minimize the possibility that the charge transfer is mediated by contaminations.

As can be seen from Fig. S2, the annealing in high vacuum can not recover the pristine surface of as exfoliated graphene, however, 6P molecules grown on the annealed devices tend to form significantly longer needle-like crystallites with clear tendency to follow crystallographic directions dictated by the symmetry of the underlaying graphene lattice. Longer annealing times, or higher annealing temperatures, or annealing in H_2/Ar atmosphere could help to restore pristine surface of graphene – yet risking to further dope the devices – and can result in unstable electrical properties due to formation of the defects with the graphene lattice and covalently bonded species.

Morphology of 6P and C_{60} films grown on graphene FETs.

Ex-situ AFM topography images of the samples have been used to determine the total volume of the deposited molecules on the active surfaces of graphene FETs. Considering the most likely bulk structures for both, 6P and C_{60} for the chosen growth conditions, the total number of molecules has been estimated. Unfortunately, under the chosen growth conditions neither 6P nor C_{60} experiences an ideal layer-by-layer growth on the surface of graphene FETs. Figure S3 presents two typical cases of the resulting growth morphologies of 6P and C_{60} layers grown on graphene FETs (T_D = 300 K).

In the case of C_{60} (Fig. S3a,b), a rather uniform film thickness was obtained across the entire surface of graphene, while on the surface of SiO_2 3D structures were found. The thickness of the grown films was expressed in the equivalent volume of the complete mono-layers considering that each layer of C_{60} should add ∼0.8 nm of the total height and that the initial height of graphene was measured prior to the growth. In the case shown in Fig. S3b, two complete mono-layers have been grown on the surface of the device, with an incomplete third and fourth layer on top. This sums up to ∼3 ML covering the entire

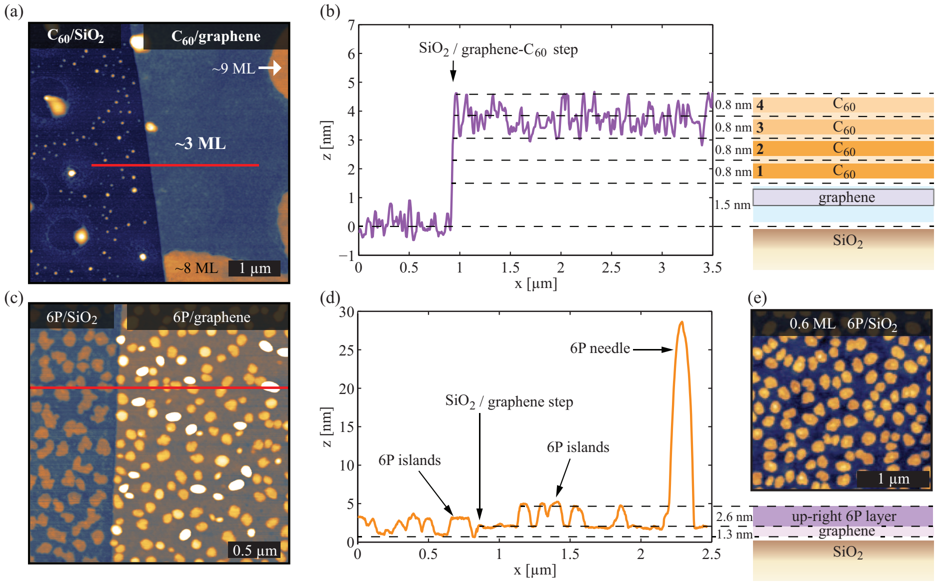

Figure S3. Morphology of C_{60} and 6P films grown on graphene FETs. (a) AFM topography image ($5\times5\ \mu m^2$, z-scale 25 nm) of a step edge between graphene channel (right) and SiO_2 substrate (left), covered with $\sim$4 ML of C_{60}. (b) Height cross-section (indicated by the solid red line in (a)), overlaid with a corresponding scheme of the interface. (c) AFM topography image ($2.5\times2.5\ \mu m^2$, z scale 10 nm) of a step edge between graphene channel and SiO_2 substrate, covered with $\sim$0.8 ML of 6P. (d) Height cross-section (indicated by the solid red line in (c)), with the corresponding scheme of the interface (bottom-right). (e) $2.5\times2.5\ \mu m^2$ (z scale 5 nm) AFM topography image of 0.6 ML of 6P covered SiO_2 of the same sample as shown in (c).

device. However, edge decorations and several higher structures have also been observed, and considering their total volume gives (4.3 $\pm$0.9) ML of C_{60} coverage for the particular case presented in Fig. S3a,b (also in Fig. 4a-c of the main manuscript).

While C_{60} can be considered as a zero-dimensional building block, 6P on the other hand will form different crystallites depending on the orientation of the molecule with the respect to the substrate plane. If the molecules are flat-lying then 3D needle-like crystallites are formed. If the molecules are nearly up-right standing ($\sim$2.6 nm), island-like crystallites grow. Both types of crystallites have the same monoclinic β-phase structure. The total volume of 6P has been considered for each sample and has been expressed as the equivalent of the complete covered mono-layers of up-right standing molecules. Figure S3c-e shows an example of 6P grown on the sample presented in Fig. 1d,e and Fig. 2a-c of the main text. In this case, 6P islands cover 25% of the device surface. However, needle-like crystallites also contribute significantly to the total volume of the deposited 6P. If these are also taken into account, the volume totally deposited would be equal to (0.8$\pm$0.2) ML. Surrounding SiO_2 had a coverage of (0.6$\pm$0.1) ML of 6P (Fig. S3e). This is in a good agreement with the total volume of 6P deposited on graphene considering that the sticking coefficient of the molecules is not the same in these two cases and that for needle-like crystallites – found on graphene – there is a larger overestimation of the volume due to the tip-sample dilation.

The calculation of the position of the Fermi level in graphene

The position of the Fermi level in graphene was calculated considering a parallel plate capacitor between graphene and SiO_2, and linear approximation of the dispersion relation of graphene. The area specific capacitance of the gate oxide was considered as $C=\varepsilon_0\varepsilon_r/d$. d stands for the thickness of the dry thermal SiO_2 ($\varepsilon_r = 3.9$). The thickness of the oxide was determined by ellipsometry measurements and was found to be $d = (80\ \pm2)\ nm$. The expected variation of the oxide thickness introduces an uncertainty of less than 2% for the determined position of the Fermi level. Thus it can be ignored, since a several times larger uncertainty is introduced by the measurements of the transfer curves ($I_{SD}(V_{SG})$). The density of the electrons in the capacitor is obtained as $n = CV_{SG}/e$. On the other hand, considering a linear approximation for the dispersion relation of graphene: $E(k) = \hbar v_F k$, where v_F stands for he Fermi velocity of electrons in graphene (10^6 m/s), the density of electrons can be expressed as: $n = {E_F}^2/\pi\hbar^2 {v_F}^2$. This gives the relation between the position of the Fermi level and the applied voltage at the gate as: $E_F = \hbar v_F((\pi\varepsilon_0\varepsilon_r/e)\cdot(V_{CNP}/d))^{1/2}$, where V_{CNP} stands for the V_{SG} which is needed to reach the minimum current in the transfer curves. Considering the known thickness of SiO_2 and the Fermi velocity in graphene yields a rather simple estimation for the position of the Fermi level as: $E_F = \pm 60.5\cdot(V_{CNP}[V])^{1/2}\ meV$.
